# Older Adult Cancer Survivors’ Functional Limitations and Determinants of Health: Evidence from the 2021 National Health Interview Survey

**DOI:** 10.3390/jcm15020856

**Published:** 2026-01-21

**Authors:** Anna Kate Autry, Zarmina Amin, Zan Gao

**Affiliations:** 1 Webb School of Knoxville, Knoxville, TN 37928, USA; 2 Department of Kinesiology, Recreation, and Sport Studies, University of Tennessee-Knoxville, 328 HPER Building, 1914 Andy Holt Avenue, Knoxville, TN 37928, USA; zamin@utk.edu

**Keywords:** aging, cancer survivorship, functional limitations, National Health Interview Survey (NHIS), physical activity

## Abstract

**Background/Objectives**: Functional limitations are common among older cancer survivors and tend to increase with age and survivorship duration. Physical activity (PA) associates with better functional outcomes, but little is known about how these associations vary as time passes post-diagnosis. This study examined how years since diagnosis, three types of physical activity, and their interactions associate with functional limitations in older cancer survivors. **Methods**: Data drawn from the 2021 National Health Interview Survey (NHIS), representing adults aged 55+ and with a prior cancer diagnosis (*n* = 9356; mean age = 72.17 ± 8.5 years), were studied. A four-item self-reported difficulty index (i.e., washing/dressing, walking one block, climbing stairs, and picking up/opening objects) was summed to measure functional limitations. PA was assessed using the items aligned with the United States PA Guidelines. Hierarchical regression was used to evaluate associations between functional limitations and years since diagnosis, vigorous physical activity, moderate physical activity, and strength training. Interaction effects of years since diagnosis and each activity type were also examined. Covariates were age, sex, BMI, and educational attainment. **Results**: Elapsed time since cancer diagnosis positively associated with functional limitations in interaction with physical behaviors, while moderate physical activity and strength training negatively associated with functional limitations. Interactions of years since diagnosis and both moderate physical activity and strength training revealed smaller increases in functional limitations. No interaction effects were observed for vigorous physical activity. **Conclusions**: Among older cancer survivors, the association between survivorship duration and functional limitations differs by engagement in moderate and resistance-based physical activity. These findings support the clinical importance of promoting sustainable, non-vigorous physical activity in long-term survivorship care.

## 1. Introduction

Cancer survivorship in the United States has increased substantially over recent decades due to improvements in early detection and treatment, with more than 18 million survivors currently living nationwide [1]. As cancer survivors age, functional limitations become increasingly salient. Older adult cancer survivors experience higher rates of mobility difficulty, reduced physical functioning, and greater impairment in activities of daily living compared with individuals without a cancer history [2,3,4]. These and other functional limitations can persist for many years after diagnosis and substantially affect survivors’ independence, quality of life, and healthcare costs [5,6,7]. Functional limitations reflect multiple biological and behavioral influences, including treatment-related toxicities and fatigue, accelerated aging, systemic inflammation, and multimorbidity [8,9,10,11,12].

Functional limitations among cancer survivors can vary considerably over time. While some cancer survivors experience gradual recovery, others exhibit progressive functional decline as long-term treatment effects accumulate [13]. Understanding whether functional limitations differ according to years since diagnosis (YSD) is therefore essential for identifying periods of heightened vulnerability across the survivorship trajectory. Additionally, physical activity (PA) has been consistently associated with fewer functional limitations in older adults [14]. Among cancer survivors, engagement in moderate-intensity aerobic activity and strength training has been linked to lower disability, improved mobility, enhanced muscle function, and reduced treatment-related side effects [15,16,17]. However, much of this evidence is derived from intervention trials or small cohort studies that may not fully represent the broader survivor population. Moreover, little is known about whether the association between PA and functional limitations varies as a function of survivorship duration. It is plausible that PA is more strongly associated with functional preservation later in survivorship, when acute treatment effects have diminished and age-related functional decline becomes more prominent.

This study extends survivorship research by demonstrating that associations between PA and functional limitations are conditional on survivorship duration in a large, nationally representative sample. Large-scale, population-level analyses linking PA, YSD, and functional limitations among older cancer survivors remain scarce. The National Health Interview Survey (NHIS) assesses functional limitations using standardized difficulty ratings for routine mobility and activities of daily living [18]. Recent updates to the NHIS incorporated multiple standardized measures of PA and functional limitations [19], providing a unique opportunity to examine these associations in a nationally representative sample. Accordingly, the purpose of this study was to examine associations between YSD, PA intensity, and functional limitations in older adult cancer survivors, while controlling for basic demographic and health characteristics. Specifically, this study evaluated: (1) whether YSD was associated with functional limitations independent of age, sex, body mass index, and education; (2) whether three forms of PA—moderate-intensity activity, vigorous activity, and strength training—were directly associated with functional limitations; and (3) whether interactions between PA type and YSD were associated with functional limitations. Clarifying these relationships may help identify when, and for whom, PA is most strongly associated with preserved functioning among older adult cancer survivors.

## 2. Materials and Methods

### 2.1. Study Design and Data Source

This cross-sectional study used data from the 2021 NHIS Sample Adult file, accessed through the Integrated Public Use Microdata Series (IPUMS) version 7.5 Health Surveys database [18]. The NHIS employs a multistage, stratified sampling design to obtain a nationally representative sample of non-institutionalized adults in the United States. The 2021 cycle was selected because it includes standardized assessments of PA and functional limitation relevant to the study aims. All data were publicly available and fully de-identified (See Appendix A); therefore, institutional review board approval was not required.

### 2.2. Participants

Participants were eligible if they were aged 55 years or older and reported at least one prior cancer diagnosis other than non-melanoma skin cancer. Exclusion criteria included age younger than 55 years, implausible PA values, current pregnancy, lack of a reported cancer diagnosis, or excessive missing responses to multiple functional limitation items or covariates. Implausible PA values were defined using logical thresholds, specifically weekly totals exceeding 1680 min per week for aerobic activity and strength/resistance training sessions exceeding 14 per week. After applying these criteria, the analytic sample included 9356 older adults with a history of cancer. Sensitivity analyses retaining these extreme PA values did not meaningfully alter regression coefficients, statistical significance patterns, or overall conclusions.

### 2.3. Measures

#### 2.3.1. Functional Limitations

Functional limitations served as the dependent variable. NHIS includes a wide set of mobility and daily functioning questions; however, many items overlap conceptually. To avoid redundancy and multicollinearity, four non-overlapping items were selected to represent distinct functional domains. Respondents reported the level of difficulty they experienced when washing or dressing, walking one block on level ground, climbing a flight of stairs, and picking up or opening small objects. Each item used a four-point scale ranging from “no difficulty” to “unable to perform”. These four items were summed to create a researcher-defined composite index, with higher scores indicating greater functional limitation. The composite demonstrated acceptable internal consistency in this study (Cronbach’s α > 0.7). Although each item is ordinal (four response categories), the summed index is commonly treated as approximately continuous in large population-based studies, enabling interpretable estimation of associations and interaction effects. Sensitivity analyses using alternative ordinal and factor-based specifications yielded substantively similar conclusions and justify the linear modeling approach. As a sensitivity check, models were re-estimated using ordinal regression treating each item as ordered categorical and using factor-score specifications derived from exploratory factor analysis; in all cases, interaction effects between years since diagnosis and PA remained directionally consistent.

#### 2.3.2. Years Since Cancer Diagnosis

YSD was defined as the difference between the NHIS survey year (2021) and the reported year of earliest cancer diagnosis, representing survivorship duration in full years. The earliest diagnosis was selected for participants with multiple cancers to reflect the maximum survivorship duration. YSD was expressed in full years, consistent with NHIS reporting conventions. Chronological age and YSD capture distinct constructs—biological aging versus survivorship duration—and were included simultaneously to estimate the unique association of survivorship duration with functional limitations while adjusting for age. Correlation between age and YSD was moderate (r = 0.107), and variance inflation factors remained below accepted thresholds (maximum VIF = 1.19), indicating acceptable collinearity.

#### 2.3.3. PA Variables

PA was assessed using NHIS items aligned with the United States PA Guidelines. Respondents reported the total weekly duration of moderate-intensity aerobic activity and vigorous-intensity aerobic activity, and the total number of weekly strength training sessions. Weekly minutes of moderate and vigorous activity were computed separately, and strength training was recorded as the number of weekly sessions. All PA variables were treated as continuous measures.

#### 2.3.4. Covariates

Age in years, sex, educational attainment, and body mass index (BMI) were included as covariates due to their established associations with physical functioning. BMI was calculated from self-reported height and weight. Age was included as a covariate alongside YSD to statistically distinguish survivorship duration effects from effects attributable to chronological aging.

### 2.4. Statistical Analysis

Hierarchical multiple linear regression was used to examine the associations among demographic factors, YSD, PA variables, and functional limitations. The dependent variable was functional limitations. Model 1 included the covariates, age, sex, education, and BMI, as baseline predictors. Model 2 added YSD. Model 3 added moderate PA, vigorous PA, and strength training frequency. Model 4 included the interaction terms between YSD and each PA variable. All continuous predictors were standardized prior to computing interaction terms to place variables on comparable scales and to reduce non-essential multicollinearity.

Although NHIS employs a complex survey design with sampling weights, strata, and clustering, unweighted regression models were used in this study. Preliminary weighted analyses incorporating interaction terms produced model instability and convergence issues. Consistent with prior NHIS-based studies examining analytic subpopulations and interaction effects, unweighted models were therefore selected to prioritize stable estimation of associations. While this approach primarily limits population-level generalizability, parameter estimates and standard errors may also be modestly affected by the absence of survey weights. Similar analytic decisions have been adopted in prior NHIS research focusing on interaction effects and subgroup inference (e.g., [2,13]).

Regression assumptions included standard diagnostic procedures. Variance inflation factors were below accepted thresholds (all VIFs < 3.5), indicating no problematic multicollinearity. Visual inspection of residual-versus-fitted plots indicated homoscedasticity, and residual distributions demonstrated acceptable normality. Model fit was evaluated using R^2^ and ΔR^2^, standardized β coefficients were reported as effect-size estimates. Because the hierarchical models represented sequential adjustments rather than independent hypothesis tests, no Bonferroni or family-wise correction was applied. Given the large analytic sample (*n* = 9356), the study had sufficient power (>90%) to detect small effect sizes in multivariable models. Analyses were conducted using IBM SPSS Statistics Version 29.0.

## 3. Results

Descriptive characteristics of the sample are summarized in Table 1. Hierarchical regression results examining associations between demographic factors, YSD, PA variables, and functional limitation scores are presented in Table 2.

Model 1 (Demographic and Health Covariates): In the initial model, older age (β = 0.244, *p* < 0.01) and higher BMI (β = 0.217, *p* < 0.01) were associated with greater functional limitations. Male sex was associated with fewer limitations compared with females (β = −0.098, *p* < 0.01), and higher educational attainment was associated with lower functional limitation (β = −0.138, *p* < 0.01). Model 1 accounted for a noteworthy proportion of the variance in functional limitations (R^2^ = 0.126).

Model 2 (Covariates + YSD): The addition of YSD to the base model did not yield a significant main effect (β = 0.015, *p* > 0.05) and resulted in only a trivial improvement in model fit (R^2^ = 0.127; ΔR^2^ = 0.001). All demographic predictors remained significant and in the same direction.

Model 3 (Covariates + YSD + PA Variables): Moderate-intensity activity (β = 0.02, *p* > 0.05), vigorous-intensity activity (β = −0.04, *p* > 0.05), and strength training frequency (β = −0.081, *p* > 0.05), were entered into a third model alongside the covariates and YSD. Results suggest no direct associations between PA and functional limitations. The inclusion of PA variables increased overall model fit (R^2^ = 0.153, ΔR^2^ = 0.026), although education was no longer statistically significant in this model. The increase in explained variance despite non-significant individual PA coefficients likely reflects shared variance and suppression effects, whereby PA variables contribute collectively rather than independently to functional limitation outcomes.

**Figure 1:**
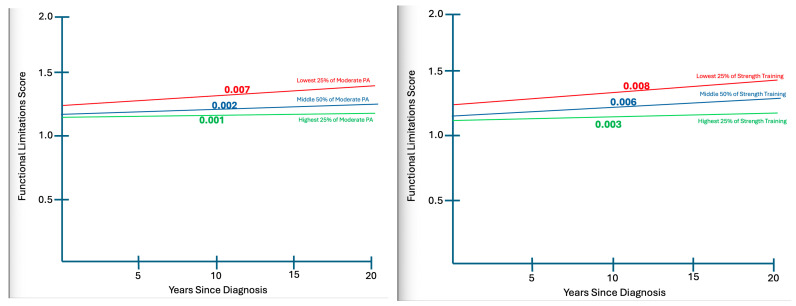
Interaction effects of YSD and PA on functional limitations.

Model 4 (Full model with Interaction Effects): The addition of interaction terms yielded a meaningful improvement in model fit (R^2^ = 0.186; ΔR^2^ = 0.033). Two interactions were statistically significant. Moderate-intensity activity moderates the association between YSD and functional limitations, such that greater moderate activity was linked to lower limitations among long-term survivors (β = −0.28, *p* < 0.05). Strength training showed an even stronger moderating effect, with higher frequency associated with fewer limitations as survivorship duration increased (β = −0.363, *p* < 0.05). The interaction between vigorous activity and YSD was not statistically significant (β = 0.205, *p* = 0.094). With interaction terms included, YSD emerged as a significant positive predictor of functional limitations (β = 0.363, *p* < 0.05). Importantly, once interaction terms are included, the YSD main effect should not be interpreted as a population-average association with functional limitations, but rather as a conditional effect evaluated at mean-centered levels of PA. The emergence of YSD as a significant predictor in Model 4 reflects its conditional association with functional limitations at varying levels of PA. When interaction terms are present, main effects represent conditional associations evaluated at mean-centered levels of the interacting variables and should not be interpreted as average marginal effects across all PA levels. To aid interpretation of these interaction effects, Figure 1 presents predicted functional limitation scores across YSD at the lowest 25%, middle 50%, and highest 25% levels of moderate PA and strength training.

As seen in Figure 1, PA types of interest, participants in the lowest 25% range demonstrate substantially more limitation (5–6%) more limitation than those in the highest quartile of participation as each decade of survivorship passes.

## 4. Discussion

This study examined the associations among YSD, PA behaviors, and functional limitations in a nationally representative sample of older adult cancer survivors. Three primary findings emerged. First, demographic factors—particularly age, sex, BMI, and education attainment—were strong and consistent predictors of functional limitations, supporting a null hypothesis that sociodemographic factors would contribute substantially to functional outcomes. Second, YSD alone was not directly associated with functional limitations when evaluated at mean-centered levels of PA, indicating that survivorship duration is not, in isolation, a reliable marker of functional limitations status. Third, although moderate and vigorous PA and strength training did not show significant direct associations with functional limitations, both moderate aerobic PA and strength training exhibited significant interactions with YSD. These interaction effects indicated that moderate-intensity aerobic PA and strength training were increasingly associated with fewer functional limitations as survivorship duration increased. In other words, the functional advantages of these PA behaviors became more pronounced among long-term survivors. By contrast, vigorous PA did not exhibit a significant interaction with YSD, suggesting that its association with function does not vary meaningfully across survivorship stages.

### 4.1. Interpretation of Key Findings

Demographic predictors demonstrated strong associations with functional limitations, aligning with prior research that advanced age, higher BMI, and lower educational attainment are linked to mobility impairments and reduced functional reserve among cancer survivors [20]. These findings confirm expectations that demographic covariates account for substantial variance in functional limitations.

Contrary to our hypothesis, YSD alone was not directly associated with functional limitations. Survivorship trajectories vary widely due to cancer type, treatment exposure, comorbidities, and socioeconomic resources, resulting in divergent long-term functional outcomes [21]. Some survivors experience functional recovery over time, while others develop persistent or late-emerging impairments linked to treatment toxicity, cardiometabolic dysregulation, or psychological burden [4]. This heterogeneity likely obscured any linear relationship between YSD and functional status.

The absence of direct associations between PA and functional limitations may reflect methodological and behavioral factors. Self-reported PA is prone to recall error and social desirability bias, often leading to misclassification and attenuation of true associations [14,22,23]. Additionally, the NHIS PA measures lack specificity about type, duration, and context, introducing further error variance. Functional benefits of PA accumulate over years, yet cross-sectional surveys capture only a snapshot of demonstrated behaviors. Older survivors also face barriers to PA engagement—including fatigue, pain, and limited access to appropriate exercise environments—which can reduce consistency and diminish observed associations [22].

The significant interactions between YSD and both moderate PA and strength training are particularly noteworthy. These findings suggest that these forms of PA become increasingly protective as survivorship progresses. Long-term survivors exhibit physiological changes resembling accelerated aging, including chronic inflammation, mitochondrial dysfunction, reduced muscle mass, and declining neuromuscular efficiency [24]. Moderate aerobic PA and strength training directly counter these mechanisms by improving mitochondrial function, preserving lean muscle mass, reducing inflammatory burden, and enhancing physical resilience [25]. These biological pathways provide a clear rationale for why moderate PA and strength training showed stronger associations with functional limitations among long-term survivors: those with the greatest vulnerability may benefit the most.

In contrast, vigorous PA did not significantly interact with YSD in abating functional limitations. Older adults, particularly those with chronic conditions or treatment-related sequelae, engage in vigorous PA infrequently, resulting in limited variability and reduced statistical power. It is also the least accurately reported PA domain, further complicating interpretation. Additionally, very high-intensity exercise may exceed physiological tolerance in older survivors, leading to poor adherence or limited benefit [26]. Moderate and resistance-based PA appear to be the most feasible and impactful modalities for this population.

These findings align with meta-analyses showing that moderate aerobic PA and resistance training improved mobility, reduced fatigue, and better functional performance in cancer survivors [27]. Combined aerobic-resistance programs appear particularly effective across treatment and survivorship stages [28]. However, most evidence originates from controlled intervention studies with limited generalizability [29,30,31]. Through our analysis of the NHIS data, this study extends those findings to a broader survivor population and highlights how different types of PA may interact with survivorship duration to influence functional outcomes in real-world contexts.

### 4.2. Reverse Causation and Alternative Explanations

Reverse causation remains a critical alternative explanation to consider. Survivors with fewer functional limitations may be more likely to engage in PA, rather than PA reducing functional decline. Prior studies have shown that functional impairment predicts lower PA participation among cancer survivors [23]. Because cross-sectional data cannot disentangle these temporal pathways, longitudinal studies are needed to clarify directionality. Accordingly, the observed interaction effects should be interpreted as conditional associations rather than evidence that PA causally preserves function.

### 4.3. Clinical and Public Health Implications

These findings hold meaningful implications for survivorship care. The observed interaction effects suggest that moderate-intensity PA and strength training may be particularly important for long-term survivors who are biologically more vulnerable to decline. Clinical guidelines, including those from the American College of Sports Medicine, emphasize the role of continued PA in reducing treatment-related symptoms and supporting functional health throughout survivorship [32]. Routine PA assessment and functional screening should be incorporated into survivorship care plans, with referrals to accessible, age-appropriate exercise programs. Public health efforts should prioritize PA programs that are safe, feasible, and tailored to older adults given the benefits of regular participation in PA for cancer survivors [33,34], including resistance training and low- to moderate-intensity aerobic PA. These findings underscore the need for longitudinal and intervention-based studies that test whether targeted moderate-intensity aerobic PA and resistance training programs can causally preserve functional independence among long-term cancer survivors.

### 4.4. Limitations and Future Directions

This study has several limitations that should be considered when interpreting the findings. First, the cross-sectional design precludes causal inference. Because preserved function may also enable greater participation in PA, reverse causation cannot be ruled out. Additionally, survivorship bias may be present, as individuals who survive longer and remain sufficiently healthy to engage in PA are likely to represent a more resilient subset of cancer survivors. Second, PA measures relied on self-reported, increasing risk of bias and underestimation of true physical behaviors. Third, the functional limitation composite, though based on standardized NHIS items, does not constitute a validated clinical measure. Fourth, key cancer-related characteristics, cancer type, stage, treatment exposure, treatment end data, and comorbidity burden, were unavailable, likely introducing residual confounding. Finally, NHIS excludes institutionalized adults, long-term care, and individuals unable to complete interviews, potentially underestimating impairment among the most vulnerable survivors.

Future research should prioritize longitudinal datasets, objective PA measurement (e.g., accelerometry), validated functional assessments, and inclusion of clinical treatment history. Examining psychosocial, behavioral, and environmental determinants of PA in long-term survivorship may further clarify mechanisms linking PA and functional health.

## 5. Conclusions

In summary, this study found that moderate-intensity PA and strength training were more strongly associated with lower functional limitations among long-term cancer survivors, despite the absence of direct effects in the overall sample. These findings underscore the potential value of sustained, feasible PA behaviors in preserving functional ability as survivorship progresses. Tailoring PA recommendations to survivorship duration may enhance the effectiveness of clinical and public health strategies aimed at maintaining independence and quality of life in older cancer survivors [35].

## Figures and Tables

**Table 1:** Demographic Characteristics of Sample (n = 9356).

Age	72.17 ± 8.50
BMI	27.78 ± 5.34
Male	44.5%
Female	55.5%
Education Level	
Less than High School Diploma/GED	733 (7.8%)
High School Graduate/GED	2281 (24.4%)
Some College/Vocational School	2695 (28.8%)
Bachelor’s degree	1961 (21.0%)
Advanced Degree	1659 (17.7%)
Unknown/Refused	27 (0.01%)

**Table 2:** Hierarchical Regression Results.

	Model 1		Model 2		Model 3		Model 4	
	B (β)	CI	B (β)	CI	B (β)	CI	B (β)	CI
Constant	−0.023	(−0.135, 0.089)	−0.026		0.112		0.020	
AGE	0.014 (0.244) **	(0.211, 0.222)	0.014 (0.241) **	(0.013, 0.015)	0.007 (0.205) **	(0.003, 0.011)	0.007 (0.213) **	(0.003, 0.011)
SEX	−0.098 (−0.100) **	(−0.118, −0.078)	−0.094 (−0.098) **	(−0.114, −0.074)	−0.086 (−0.169) *	(−0.151, −0.020)	−0.083 (−0.163) **	(−0.148, −0.018)
EDU	−0.001 (−0.138) **	(−0.001, 0.000)	−0.001 (−0.133) **	(−0.001, 0.000)	0.000 (0.064)	(0.000, 0.000)	0.000 (0.062)	(0.000, 0.000)
BMI	0.020 (0.217) **	(0.018, 0.022)	0.020 (0.219) **	(0.018, 0.022)	0.021 (0.348) **	(0.013, 0.029)	0.021(0.343) **	(0.013, 0.028)
YSD	-		0.001 (0.015)	(0.000, 0.002)	0.001 (0.039)	(−0.002, 0.004)	0.009 (0.363) *	(0.002, 0.015)
MOD (min/wk)					0.005 (0.020)	(−0.027, 0.037)	0.045 (0.179)	(−0.003, 0.093)
VIG (min/wk)					−0.012 (−0.040)	(−0.049, 0.025)	−0.046 (−0.158)	(−0.099, 0.007)
STR (ses/wk)					−0.031 (−0.081)	(−0.076, 0.015)	0.045 (0.118)	(−0.032, 0.121)
YSD × MOD							−0.001 (−0.280) *	(0.000, 0.000)
YSD × VIG							−0.001 (0.205)	(0.000, 0.000)
YSD × STR							−0.002 (−0.363) *	(−0.003, 0.000)
R^2^	0.126		0.127		0.153		0.186	
Change in R^2^			0.001		0.026		0.033	

Notes: Unstandardized (B) and standardized regression coefficients (β) are reported. All continuous predictors were standardized prior to analysis. Confidence intervals correspond to unstandardized coefficients; standardized β values are reported for comparability of effect sizes. When interaction terms are included, main effects represent conditional associations evaluated at mean-centered levels of the interacting variables rather than average marginal effects. Model 1 includes demographic covariates (age, sex, education, BMI). Model 2 adds YSD. Model 3 adds PA variables (moderate-intensity aerobic activity, vigorous-intensity aerobic activity, and strength training frequency). Model 4 adds interaction terms between YSD and each PA variable. No correction for multiple testing was applied because interaction terms reflect theory-driven, planned contrasts within a hierarchical modeling framework. * *p* < 0.05; ** *p* < 0.01.

## Data Availability

Data can be freely downloaded from www.ipums.org.

## Appendix A

**Variable Definitions and NHIS Variable Codes**

Age: Age in years [IPUMS/NHIS code: AGE].

Sex: Biological sex at birth, 0 = female, 1 = male [IPUMS/NHIS code: SEX].

Education Attainment: categorical, with 1 = grades 1–11, 11 = terminal or professional degree [IPUMS/NHIS code: EDUC].

BMI: Body Mass Index, calculated from publicly released height and weight variables [IPUMS/NHIS code: BMI].

Years Since Diagnosis: Years since a patient’s initial cancer diagnosis (calculated as survey year minus diagnosis year, in full years) [IPUMS/NHIS code: BIRTHYR, CNXXXXAG].

Moderate PA: Moderate PA in minutes per week [IPUMS/NHIS code: MOD10DMIN].

Vigorous PA: Vigorous PA in minutes per week [IPUMS/NHIS code: VIG10DMIN].

Strength training: Strength/resistance training in sessions per week [IPUMS/NHIS code: STRONGFWK].

(DV) Functional Limitations: Four-item index measure calculated as the sum of four items:

DP1: Amount of difficulty washing or dressing (1 = none, 4 = extreme) [IPUMS/NHIS code: LAWASHDRRESDIF].

DP2: Amount of difficulty walking one block on level ground (1 = none, 4 = extreme) [IPUMS/NHIS code: WALKDIF1BL1].

DP3: Amount of difficulty walking up or down 12 steps (1 = none, 4 = extreme) [IPUMS/NHIS code: WALKDIF12SD1].

DP4: Amount of difficulty picking up objects or opening bottles (1 = none, 4 = extreme) [IPUMS/NHIS code: LAHANDDIF].
